# Innate Immune Program in Formation of Tumor-Initiating Cells from Cells-of-Origin of Breast, Prostate, and Ovarian Cancers

**DOI:** 10.3390/cancers15030757

**Published:** 2023-01-26

**Authors:** Sen Han, Xueqing Chen, Zhe Li

**Affiliations:** 1 Division of Genetics, Brigham and Women’s Hospital, Boston, MA 02115, USA; 2 Department of Medicine, Harvard Medical School, Boston, MA 02115, USA

**Keywords:** tumor-initiating cell, cancer stem cell, cellular origin, cell-of-origin, breast cancer, prostate cancer, ovarian cancer, innate immune program, Toll-like receptors, immune-checkpoint-blockade-based immunotherapy

## Abstract

**Simple Summary:**

Tumor-initiating cells, also known as cancer stem cells, are a subset of cancer cells in a tumor that sustain the tumor and are often responsible for therapy resistance and relapse. Developmentally, they are evolved from the cellular origin of their corresponding cancer type and, as a result, may inherit some expression programs from the cellular origin. This review aims to summarize data from the literature showing that several hormone-related cancers (i.e., breast, prostate, and ovarian) have a preferred luminal progenitor origin. These luminal progenitors express a common innate immune program (e.g., Toll-like receptors and their associated genes). Tumor-initiating cells originated from such luminal progenitors may inherit this program, which may contribute to their formation via activation of Toll-like receptor pathways and crosstalk with immune cells (e.g., macrophages). We propose a potential strategy to eliminate such tumor-initiating cells by enhancing immunotherapy via further activation of their inherited innate immune pathways.

**Abstract:**

Tumor-initiating cells (TICs), also known as cancer stem cells (CSCs), are cancer cells that can initiate a tumor, possess self-renewal capacity, and can contribute to tumor heterogeneity. TICs/CSCs are developed from their cells-of-origin. In breast, prostate, and ovarian cancers, progenitor cells for mammary alveolar cells, prostate luminal (secretory) cells, and fallopian tube secretory cells are the preferred cellular origins for their corresponding cancer types. These luminal progenitors (LPs) express common innate immune program (e.g., Toll-like receptor (TLR) signaling)-related genes. Microbes such as bacteria are now found in breast, prostate, and fallopian tube tissues and their corresponding cancer types, raising the possibility that their LPs may sense the presence of microbes and trigger their innate immune/TLR pathways, leading to an inflammatory microenvironment. Crosstalk between immune cells (e.g., macrophages) and affected epithelial cells (e.g., LPs) may eventually contribute to formation of TICs/CSCs from their corresponding LPs, in part via STAT3 and/or NFκB pathways. As such, TICs/CSCs can inherit expression of innate-immunity/TLR-pathway-related genes from their cells-of-origin; the innate immune program may also represent their unique vulnerability, which can be explored therapeutically (e.g., by enhancing immunotherapy via augmenting TLR signaling).

## 1. Brief Summary of TICs/CSCs

Tumor-initiating cells (TICs), also known as cancer stem cells (CSCs), are defined as a subpopulation of cancer cells that can initiate a tumor, possess self-renewal capacity, and can contribute to heterogeneous lineages of cancer cells that comprise the tumor [1]. CSCs were originally identified in human acute myeloid leukemia (AML) by the pioneering work of John Dick and colleagues [2]. They separated AML cells from patients based on cell surface markers, such as CD34 and CD38. By transplanting different subsets of AML cells to immunodeficient NOD/SCID mice, they found that only AML cells within the CD34^+^CD38^−^ fraction were capable of recapitulating human AML in NOD/SCID recipients; these were referred to as leukemic stem cells (i.e., CSCs for AML). Later, similar approaches were used to identify CSCs in solid tumors, such as breast cancer [3,4], brain tumor [5], colon cancer [6,7,8], prostate cancer [9,10,11,12], and ovarian cancer [13,14,15,16]. Due to the nature of the transplantation-based approach, CSCs are functionally defined as a subset of cancer cells capable of initiating tumor growth in recipient mice; thus, they are often referred to as TICs as well.

As the transplantation-based approach in mice only selects those tumor cells that can grow in the mouse microenvironment, it may not necessarily reflect the behavior of all types of cancer cells in their native habitats, which raises a concern as to whether TICs/CSCs defined in this way really exist in intact tumors. For instance, TICs/CSCs in human melanoma defined based on xenotransplantation were initially reported to be rare [17]. However, by modifying the xenotransplantation assay conditions, it was shown later that TICs/CSCs were much more common in human melanoma [18]. Nevertheless, several lines of evidence based on other approaches have provided further support for existence of TICs/CSCs: e.g., in 2012, by using genetic marking, lineage tracing, and clonal analysis approaches, several studies demonstrated the existence of CSCs during unperturbed solid tumor growth. These include a study for squamous skin cancer in which it was found that CSC populations with different properties existed in benign papilloma and invasive squamous cell carcinoma [19]. CSCs in benign papilloma are rare, mirroring the composition, hierarchy, and fate behavior of normal skin tissue; in contrast, CSCs in invasive squamous cell carcinoma are more common [19], consistent with the notion that CSCs are not necessarily a rare subpopulation of cancer cells in a tumor and also suggesting that frequency of CSCs is cancer-stage-dependent [18,19]. In another study, it was found that tumor cells expressing intestinal stem cell marker LGR5 are the CSCs to fuel growth of intestinal adenomas [20]. In glioblastoma, it was shown that a relatively quiescent subpopulation of endogenous glioma cells exhibited characteristics of CSCs to sustain long-term tumor growth through production of transient populations of highly proliferative tumor cells [21]. More recently, in human pancreatic ductal adenocarcinoma (PDAC), by using a novel marker-free lineage tracing approach coupled with quantitative modeling of tumor expansion in xenografts, it was found that all PDAC cells exhibited clonogenic potential in vivo and that the stromal microenvironment played a dominant role in defining their clonogenic activity [22]. A caveat for this study is that the data were largely based on PDAC cell lines in a xenograft setting. Last, by high-throughput sequencing analysis of human tumors, numerous DNA mutations have been identified in tumor cells that can serve as “barcodes” for lineage analysis of a tumor (i.e., tumor cells carrying the same mutations may have a common cellular origin). Interestingly, in a study analyzing the natural histology and clonal evolution of human breast cancers based on sequencing data, it was implied that there exists a long-lived, quiescent cell lineage (referred to as “the most-recent common ancestor (MRCA)”) capable of substantial proliferation upon acquisition of enabling genomic changes, thus supporting the concept of TICs/CSCs and their existence in intact tumors [23].

Although it remains controversial, the concept of TICs/CSCs is important for recognizing a subpopulation of cancer cells within a tumor that has clinical importance as these are often the type of cancer cells resistant to chemotherapeutics and that contribute to cancer relapse and, therefore, also represent the ultimate therapeutic target [24].

## 2. Formation of TIC/CSC from the Cellular Origin of Cancer

**Figure 1:**
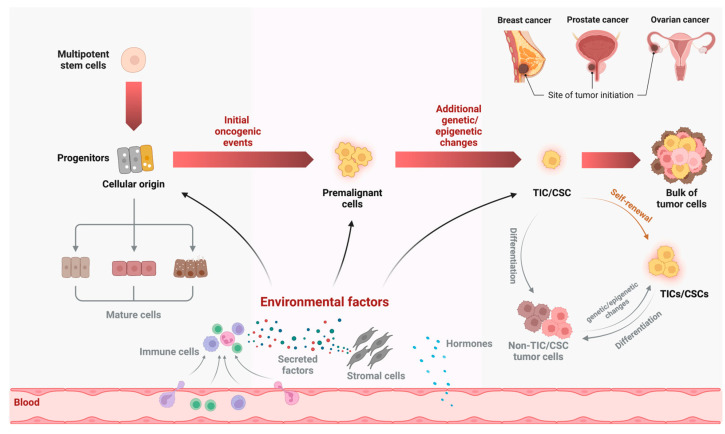
Schematic diagram depicting the developmental link between tumor-initiating cells (TICs)/cancer stem cells (CSCs) and their cellular origins (normal cells). In breast, prostate, and ovarian cancers, luminal progenitors in their corresponding tissues/organs (in the case of ovarian cancer, fallopian tube (FT)) are the preferred cellular origins.

The term “TIC” can often cause confusion with other commonly used terms, such as the cellular origin (or cell-of-origin) of cancer. Cellular origin of cancer is a population of normal cells in a tissue or organ that, with enabling initiating oncogenic events, evolve to eventually become cancer cells. In the literature, cancer-initiating cell, defined as a normal cell that receives the first cancer-causing mutations [25], is the same as the cellular origin/cell-of-origin of cancer described here. In contrast, at least for the purpose of this review, TICs refer to CSCs or a subpopulation of cancer cells in a tumor that possess stem-cell-like properties (i.e., “stemness”). Developmentally, TICs are derived from their corresponding cellular origins (normal cells) via genetic/epigenetic changes and interaction with the microenvironment and through clonal evolution (Figure 1). Because of this developmental connection, the intrinsic gene expression program in the cellular origin of cancer may also make a significant contribution to the properties of their corresponding TICs, which can be explored therapeutically (see below).

## 3. Luminal Progenitors as Cellular Origins of Breast, Prostate, and Ovarian Cancers

Cellular origins of most human cancer types are difficult to determine. For example, cells-of-origin of several hormone-related cancers, such as breast, prostate, and ovarian, have been topics of long-standing debate. Breast cancer comes from transformation of mammary epithelial cells (MECs), which include estrogen receptor (ER)^+^ luminal cells, ER^−^ luminal cells, as well as ER^−^ basal cells. Based on the cleared mammary fat pad transplantation assay, it was determined that basal MECs possess mammary stem cell (MaSC) activity and are capable of producing both basal and luminal MECs upon transplantation [26,27]. Due to the multipotency nature of MaSCs, it was initially thought that MaSCs/basal MECs are the cells-of-origin of most breast cancers [28,29]. However, more recent experimental evidence demonstrated that most breast cancers may come from transformation of luminal MECs [30,31]. Luminal progenitors (LPs) are committed progenitor cells in the luminal lineage that give rise to mature luminal cells upon their differentiation [32,33]. Among them, ER^−^ LPs are long-lived progenitor cells that give rise to milk-producing alveolar luminal cells during pregnancy and lactation (i.e., alveolar progenitors) [34,35]. Evidence from both human breast tissue and mouse modeling studies demonstrated that basal-like breast cancers (BLBCs), particularly those developed in *BRCA1* mutation carriers, originate from such LPs rather than from basal MECs [36,37,38,39]. This was further supported by our recent single-cell study in which we showed selective expansion of the alveolar LP subpopulation upon induced BRCA1 loss in a novel mouse model of BRCA1-deficient BLBC [40]. In addition, we demonstrated that mammary tumors developed in the mouse mammary tumor virus-polyoma middle T antigen (*MMTV-PyMT*) transgenic model (a mouse model for luminal B subtype of human breast cancer [41]) may have an LP origin as well [35].

Prostate epithelial cells (PECs) include luminal and basal cells (as well as a rare population of neuroendocrine cells) [42]. Based on the renal capsule reconstitution assay, it was shown initially that basal PECs exhibited multipotent prostate stem cell activity and could serve as cells-of-origin of most, if not all, prostate cancers [43,44]. However, human prostate cancer is luminal in nature and its progression to the advanced stage is characterized by a progressive loss of basal cells [45]. If prostate cancer originates from basal PECs, such cells would need to differentiate into luminal PECs first. In fact, by lineage tracing, several studies provided convincing evidence to support that luminal PECs could also serve as cells-of-origin of prostate cancer, leading to development of more aggressive prostate cancer [46,47]; in these models, although prostate cancer could also initiate from basal cells, such cancer exhibited longer latency due to a need for basal cells to differentiate into luminal cells first. In the renal capsule reconstitution assay, luminal PECs can be produced via differentiation from basal PECs [48]; however, in unperturbed adult prostates, the luminal lineage appears to be self-sustained by its own progenitor cells [46,49,50,51]. Existence of multipotent or unipotent human and mouse prostate LPs was supported by the recently developed organoid culture system [52,53,54,55,56]. Furthermore, several recent studies based on single-cell analysis have also demonstrated the presence of LPs in mouse or human prostate [55,57,58,59]. Overall, it appears that, although prostate cancer can originate from both basal and luminal PECs, luminal cells (particularly LPs) are the preferred cellular origin of prostate cancer, including castration-resistant prostate cancer (CRPC) [46,47,50,51,55,60].

Ovarian cancer, as the name suggests, was initially thought to originate from epithelial cells in the ovary, referred to as ovarian surface epithelial (OSE) cells [61,62]. However, more recent evidence suggested that the cellular origin of most epithelial ovarian cancers (EOCs), particularly the most common type, serous ovarian carcinoma, is the epithelial cell of nearby fallopian tube (FT) tissues (oviduct in mouse) [63,64,65,66,67,68]. The FT epithelium is composed of two types of FT epithelial (FTE) cells, including secretory cells and ciliated cells [69]. FTE cells are positive for Keratin 8, which is a pan-luminal marker, so they are “luminal” cells as well [70]. By lineage tracing, it was shown that FTE secretory cells have stem/progenitor cell activity and can give rise to ciliated cells, which are considered as more differentiated FTE cells [69]. It is now believed that most serous ovarian cancers originate from FTE secretory cells, particularly those in the fimbrial region of the FT (i.e., the distal region of the FT closer to the ovary) [71,72]. While both FTE and OSE cells could serve as cells-of-origin of ovarian cancer in experimental settings, their corresponding ovarian cancer types exhibited different disease latencies and therapeutic responses [73,74]. Importantly, by single-cell analysis and comparison of expression signatures, we showed recently that a subset of FTE secretory cells expressing stem/progenitor cell-related genes resemble LPs in the mammary gland at the molecular level (whereas OSE cells resemble basal MECs) [75], suggesting that they may represent LP-equivalent cells in the FT epithelium. Together, it seems that, in breast and prostate cancers, there is convincing evidence to support that the LP subpopulation in their corresponding epithelium may be the preferred cellular origin; this notion may also hold true in ovarian cancer, but more studies are needed to further demonstrate it.

## 4. Common Innate Immune Program in LPs

Transcriptome analyses of mouse and human MEC subpopulations revealed expression of multiple genes related to the innate immune pathways, particularly the Toll-like receptor (TLR) signaling pathways (e.g., genes encoding CD14, LBP, TLRs) in mammary LPs [76]. TLRs are a group of pattern recognition receptors (PRRs) in the innate immune system to recognize pathogen-associated molecular patterns (PAMPs) and endogenous damage-associated molecular patterns (DAMPs) [77]. In TLR pathways, lipopolysaccharide (LPS), a major component of Gram-negative bacteria, is the ligand for several TLRs (e.g., TLR4). CD14 binds LPS in the presence of lipopolysaccharide-binding protein (LBP) and plays a key role to load LPS onto the TLR4/MD2 (MD2 also known as LY96) complex. Activation of the TLR4/MD2 complex triggers downstream signaling cascades (e.g., activation of NFκB signaling, leading to production of proinflammatory cytokines) [78,79]. Expression of the TLR4/MD2 complex has been found in mammary LPs, and, importantly, treatment of LPs with TLR ligands led to enhanced growth of mammospheres, suggesting that these progenitor cells could directly sense and respond to microbial products [80]. In addition to sensing bacterial infection, other TLRs, such as TLR3, an endosomal TLR, can sense double-stranded RNA (dsRNA; e.g., from viral infection) [81], which eventually leads to production of mainly type I interferons (IFNs) [82].

Intriguingly, in our recent study of single-cell analysis of FT cells, we found that FTE secretory stem/progenitor cells resemble mammary LPs, and the similarity in their transcriptomes is in part due to their common expression of innate-immunity/TLR-pathway-related genes (e.g., *Lbp*, *Cd14*) [75]. This expression pattern (of immune-related genes) is also observed in human FT secretory progenitor cells [83].

In the prostate, by single-cell analysis, we identified a LY6D^+^ progenitor subpopulation in the luminal lineage; these progenitors represent multipotent and/or unipotent LPs inherently resistant to androgen deprivation and with regenerative capacity and can serve as cells-of-origin for prostate cancer initiation and progression to CRPC [55]. LY6D^+^ prostate LPs are consistent with several types of prostate LP subpopulations defined in other studies, including those by lineage tracing [49,50,51] or single-cell analysis [57,58,59]. By expression analysis, we found that innate-immunity/TLR-pathway-related genes are also expressed in these prostate LPs [55]. Of note, the inflammation/immune-related gene signature has also been observed across multiple mouse and human prostate LP datasets from another study [84].

Why do progenitors for luminal MECs, luminal PECs, and FTE cells express common innate-immunity/TLR-pathway-related genes? One possibility is that all these organs/tissues are susceptible to bacterial and/or viral infection (e.g., mastitis in the breast, prostatitis in the prostate, and salpingitis in the FT) and expression of innate immune genes in LPs that possess regenerative capacity may link the inflammation-related tissue damage to their corresponding epithelial tissue repair program, a process that may contribute to development of their corresponding cancer types. In support of this, polymorphisms in TLR genes or TLR expression have been found associated with these cancers; for instance, in breast cancer, polymorphisms in *TLR3*, *5*, and *9* genes may either increase breast cancer risk or play protective roles [85,86,87]. Genetic variations in candidate genes involved in TLR or its downstream NFκB pathways may also be associated with breast cancer risk [88]. Furthermore, expression of TLR4 and its downstream adapter protein MyD88 was found to be significantly higher in breast cancer than adjacent normal tissues, which was associated with poor prognosis [89,90]. In prostate cancer, polymorphisms in *TLR4* were reported to be associated with prostate cancer risk in several studies [91,92,93,94,95], although no significant association was also found in other studies [96,97]. In ovarian cancer, high expression of TLR4 and MyD88 was found to predict poorer overall survival in patients with EOCs [98]. A polymorphism in *TLR4* was also found recently to be associated with increased ovarian cancer risk [99]. Of note, TLR pathways have dichotomous roles in cancer development [100]. They can inhibit cancer initiation by activating innate immune reactions (and, subsequently, adaptive immune responses), leading to elimination of premalignant cells; however, if TLR-induced inflammation persists, chronic inflammation and TLR-related tissue repair can promote cancer development. Thus, any association of TLRs with clinical outcomes is likely to be cancer-type/context-dependent.

## 5. Presence of Microbes in Normal and Cancerous Breast, Prostate, and FT Tissues

It has been well studied that several human viruses (e.g., human papillomavirus (HPV), hepatitis B virus (HBV), Epstein–Barr virus (EBV)) can directly cause cancer initiation via infection of the cellular origin of their corresponding cancer type, in part due to expression of viral oncogenes and/or induction of host proto-oncogenes [101]. In addition, microbe infection (e.g., bacteria, viruses) can also contribute to cancer development indirectly via activation of innate immune pathways and induction of an inflammatory microenvironment. For instance, *Helicobacter pylori* (*H. pylori*) infection is associated with development of gastric cancer due to interactions of *H. pylori* PAMPs with PRRs located on immune and gastric epithelial cells and subsequent activation of the innate immune program [102]. Although breast, prostate, and ovarian cancers are not typical cancer types known to be associated with an infection, microbes (e.g., bacteria, fungi, and viruses) may, nevertheless, play important roles in their development from their cellular origins.

### 5.1. Microbes in Breast Tissue and Cancer

Breast tissue and milk used to be thought as sterile but are now known to contain a diverse and unique microbiome [103]. Mastitis is an inflammation of breast tissue that may involve infection and most commonly affects women who are breast-feeding. Although it remains controversial, there are some epidemiological studies showing a link between mastitis and higher risk of developing breast cancer [104,105]. With advances in sequencing technology, microbiota profiling has become feasible. By using 16S-rRNA-amplicon-sequencing-based microbiome analysis, a previous study showed that bacteria were indeed present in breast tissues, and, intriguingly, bacterial profiles in normal adjacent breast tissues from women with breast cancer are different from those in breast tissues from healthy women [106]. In particular, bacteria that had the ability to cause DNA damage in vitro were found to be more abundant in adjacent breast tissues from breast cancer patients, whereas those with anticarcinogenic properties were more abundant in normal breast tissues, raising the possibility that the breast microbiota may modulate the risk of developing breast cancer [106]. In breast cancer patients, compared to the adjacent breast tissues, breast tumors have higher bacterial load and richness (i.e., number of bacterial species) [107,108]. Overall, it appears that these differences are tissue/cancer-stage-specific (e.g., from breast tissues in healthy women to breast tissues adjacent to tumors and to breast tumors, there are increasing numbers and species of bacteria [108]). Since these human tissue studies are correlations in nature, whether microbiota in breast tissues play any direct contribution to breast cancer development is unclear. Studies in experimental models may help to elucidate this. For instance, a recent study showed that tumor-resident microbiota could promote metastatic colonization of mammary tumor cells in an *MMTV-PyMT* transgenic mouse model [109]. Thus, microbes (e.g., bacteria) are present in both normal and cancerous mammary tissues and may affect breast tumorigenesis in at least some stages of its development.

In addition to bacteria, there is also evidence showing a potential relationship of viral infection with breast cancer. Infection with human cytomegalovirus (CMV) is common in adults in developed countries (~40–70%), and this virus was shown to preferentially infect breast cancer cells with elevated expression of platelet-derived growth factor receptor-α (PDGFRα) and fibroblasts (which have high levels of PDGFRα expression); infection of PDGFRα^+^ fibroblasts raised the possibility that human CMV infection could affect the tumor microenvironment, leading to a more inflammatory milieu [110]. Previous research also reported some conflicting data of the potential roles of HPV, EBV, or MMTV in breast cancer, but the precise roles of these viruses in breast tumorigenesis remain unclear [111,112].

### 5.2. Microbes in Prostate Tissue and Cancer

Similar to breast tissue, the urinary tract in males, which goes through the prostate, was traditionally considered as a sterile body niche but is now well recognized as a reservoir of bacteria [113,114]. Urinary tract infections (UTIs) are mainly due to bacterial infections but may also be caused by fungi or viral infections [113,114]. Acute bacterial prostatitis is an acute bacterial infection of the prostate gland that is mainly caused by ascending urethral infection or intraprostatic reflux [115]. Acute bacterial prostatitis is most frequently caused by *Escherichia coli* (Gram-negative), followed by *Pseudomonas aeruginosa* and *Klebsiella*, *Enterococcus*, *Enterobacter*, *Proteus*, and *Serratia* species [116,117,118,119]. Chronic bacterial prostatitis is defined as recurrent UTIs with the same organism in prostatic secretions during asymptomatic periods [120]. The most common pathogenic agents of chronic bacterial prostatitis are also *Escherichia coli* or other Gram-negative Enterobacteriaceae [121].

As the urinary tract is considered the main route for potential bacterial infections that could affect prostate cancer development (e.g., by influencing chronic inflammation observed in the prostate), urinary microbiome was profiled in men with or without prostate cancer and the study found a prevalence of proinflammatory bacteria and uropathogens in the urinary tract of men with prostate cancer [122]. Microbiome profiling has also been performed in the expressed prostatic secretions (EPS) of patients with prostate cancer or benign prostatic hyperplasia (BPH); in this study, a cluster of bacteria, most of which are Gram-negative, was shown to be abundant in EPS from patients with prostate cancer [123]. In a third approach, cancerous prostate tissue biopsies were subjected to whole-genome sequencing directly, and, by metagenomic analysis, it was found that many common bacterial genera could be detected in prostate tissues, with a predominance for *Proteobacteria* [124]. Men from Africa or with African ancestry have elevated risks of developing lethal prostate cancer, and, intriguingly, this study found that there was increased bacterial content and richness within the African versus non-African samples, raising the possibility that oncogenic transformation driven by bacterial infection within the prostate microenvironment may be contributing to the aggressive disease presentation in African samples [124].

In addition to bacteria, viruses may also contribute to prostate cancer development. For instance, HPV can immortalize normal human PECs, and there is strong evidence supporting the association of HPV infection with increased risk of prostate cancer [125,126,127]. Other viruses, such as CMV or Polyoma viruses, may also play a role in prostate cancer, but the association is not significant [126].

### 5.3. Microbes in FT Tissue and Ovarian Cancer

With improvements in bacterial detection, the theory of the sterile female upper reproductive tract (URT) has been frequently challenged in recent years [128]. Similar to mammary and prostate glands, it is now believed that the URT in the female, which includes the FT and ovary, is also unlikely to consist of sterile structures [129]. By 16S bacteria rRNA gene analysis, it was found that bacteria indeed exist in the URT, and there were significant differences in the microbiome of FT versus ovary, as well as the proximal versus distal (fimbriae) region of the FT [129]. The latter is important as the distal region of the FT is thought to be the origin of most serous ovarian carcinomas [63,64,65,66,67,68]. In another study, by 16S rRNA high-throughput sequencing analysis, the diversity and composition of the microbiota from ovarian cancer tissues and normal distal FT tissues were compared; it was concluded that microbial composition change might be associated with the process of ovarian cancer development from the distal region of the FT [130]. As local inflammation may participate in initiation and continuation of ovarian cancer [131], the microenvironment of the FT may contribute to ovarian cancer initiation before the cancer spreads to the ovary and beyond.

The relationship of viral infection and ovarian cancer initiation is also a novel focus in clinical research. There were some studies showing an association of CMV and HPV with EOC [132,133,134]. Other studies reported no significant association but still speculated a potential role of HPV infection in ovarian carcinogenesis [135,136]. More evidence is needed to illustrate the link and mechanism.

## 6. Immune Programs in Formation of TICs/CSCs

The studies summarized above demonstrate the presence of microbes such as bacteria in mammary and prostate glands as well as FTs. A common theme appears to be the association of dysbiosis (i.e., an imbalance in the microbiota, a term originally from the study of gut microbiota [137]) with the neoplastic transformation process of their corresponding cancer type. In particular, there is often an overrepresentation of Gram-negative bacteria associated with initiation of these epithelial cancers, which is likely to be driven by LPs (as their cellular origins). As discussed above, LPS is a major component of Gram-negative bacteria and can trigger TLR pathways (e.g., TLR4). TLR4 signaling can use either MyD88-dependent or -independent downstream pathways: in the MyD88-dependent pathway, activated TLR4 uses MyD88 as an adapter to eventually activate NFκB, leading to production of proinflammatory cytokines; in the MyD88-independent pathway, activated TLR4 uses TRIF (also known as TICAM1) as the adapter, eventually leading to IRF3 activation and production of type I IFNs [79]. In breast, prostate, and ovarian cancers, studies using their corresponding cancer cell lines or primary cancer cells from patients all provided evidence to support that LPS could promote production of proinflammatory cytokines from cancer cells by signaling through TLR4 and MyD88 [77,138,139]. Of note, TLRs can also be activated by DAMPs released from damaged and/or necrotic tissues (e.g., TLR4 and TLR2 can be activated by HMGB1) [77]. Thus, the fact that LPs in breast, prostate, and FT tissues are strongly linked with the microbiome and innate-immunity/TLR-related programs provides a potential mechanistic link for involvement of microbe and immune programs in initiation and/or progression of these cancer types.

Recent studies have demonstrated a prominent association between cancer stemness (i.e., the stem-cell-like properties of TICs/CSCs) and immunity [140,141]. As discussed above, chronic inflammation in the breast, prostate, or FT tissues, in part due to the presence of Gram-negative bacteria, could trigger the innate immune program in LPs, leading to activation of the NFκB pathway and production of proinflammatory cytokines, which could attract and recruit macrophages. Macrophages have been shown as important mediators of tumor immunosurveillance [142,143]. As key innate immune cells in the mammary gland, macrophages have been shown to play dual roles in development and remodeling of MECs [144]. Macrophages have at least two activation states (i.e., polarization states [145]): the classically activated M1 state, in which they are proinflammatory and are cytotoxic against microbes and tumor cells; the alternatively activated M2 state, in which they play a role in homeostatic mechanisms that terminate inflammatory responses and promote wound healing and tissue remodeling [145,146,147]. Although M1/2 may be an oversimplified description of macrophage functional states, they at least provide a framework for understanding different roles of macrophages. TLR signaling can negatively affect cancer initiation by activating innate/adaptive immune reactions, leading to elimination of mutated or infected cells. However, if TLR-induced inflammation persists, chronic inflammation can promote cancer progression, in part due to polarization of macrophages from the M1 to the M2 state (and subsequent or accompanying changes in other immune cells). In addition to macrophages, other immune cell subpopulations, such as myeloid-derived suppressor cells (MDSCs) and certain subsets of T cells (e.g., Th17 T helper cells in ovarian cancer [148,149] and regulatory T cells in breast cancer [150]) can also contribute to formation of TICs/CSCs and/or reinforcing their stemness [140,141].

**Figure 2:**
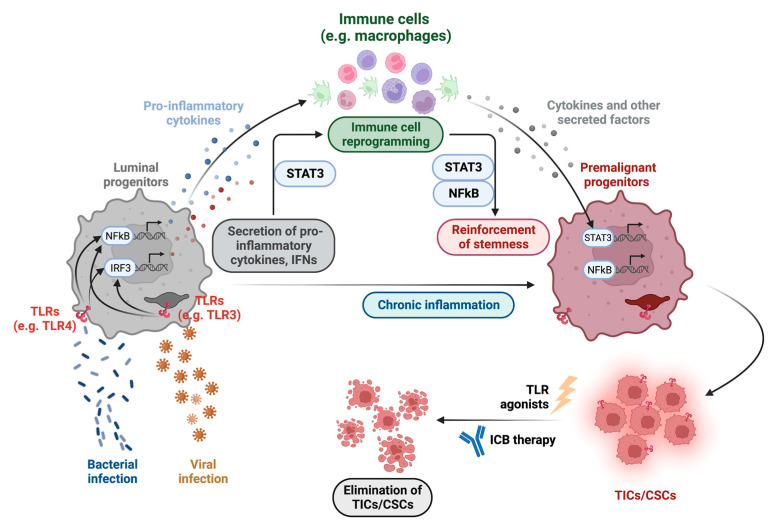
Schematic diagram depicting the potential role of innate immune program/Toll-like receptor (TLR) pathways in evolution of TICs/CSCs from their corresponding cellular origins (i.e., luminal progenitors in the breast, prostate, and FT).

The stemness of TICs/CSCs is regulated by several key pathways, among which the STAT3 pathway is an important one as this pathway can not only upregulate expression of stemness-related genes potently but also activate other stemness-promoting pathways (e.g., NFκB pathway) [141]. In fact, STAT3 is one of the most important transcription factors to regulate both stemness and innate immunity. TICs/CSCs can contribute to macrophage polarization to M2 via the STAT3 pathway in breast cancer [151] or the NFκB pathway in ovarian cancer [152] (Figure 2). In return, macrophages can contribute to formation of TICs/CSCs via these two pathways as well [153,154,155] (Figure 2). This can be achieved by inducing a positive feedback loop to reinforce the stemness of TICs/CSCs through a panel of TIC/CSC-supporting cytokines, such as IL-6, IL-8, or WNT5B [141].

In addition to recruiting various immune cells, TLR pathways also play an important role in maintaining tissue homeostasis by regulating inflammatory and tissue repair responses to injury, a process that can contribute to cancer development [156]. In various epithelial tissues, multiple lines of evidence support that expression of TLRs in epithelial cells not only allows them to detect pathogen-derived products and trigger innate and adaptive immunity but also coordinates repair of epithelial cell injury: e.g., (1) in intestinal epithelial cells, it has been shown that TLR4- and MyD88-dependent signaling induces COX2 expression, which mediates generation of PGE2 and production of growth factors, leading to intestinal epithelial proliferation [157]; (2) TLRs can also provide signals to promote survival of epithelial cells under stress conditions (e.g., through the TLR4-MyD88-NFκB-COX2 cascade in normal and premalignant colon cells [158]); (3) wound healing was impaired in *Myd88*-deficient mice [159]; (4) deletion of MyD88 or TLR2 impaired intestinal epithelial regeneration and decreased mammary epithelial repopulating unit frequency [160].

## 7. Perspective of Targeting TICs/CSCs by Enhancing Immunotherapy

As discussed above, immune mechanisms likely play key roles in development of TICs/CSCs from their cellular origins. By gene expression analysis, it was found that there is an association between cancer stemness and immune suppression in many types of solid tumors [161]. Notably, recurrent negative correlations to stemness were observed among major immune cell subpopulations that possess antitumor properties, including CD8^+^ T cells, natural killer (NK) cells, and B cells [161]. Crosstalk between TICs/CSCs and immune cells, especially myeloid cells and T cells, also enables TICs/CSCs to shape a specific immunosuppressive tumor microenvironment (TME) that facilitates tumorigenesis, metastasis, and drug resistance [151,162,163]. Thus, harnessing the immune system and reversing TME to an anti-tumor state might represent a promising strategy to target TICs/CSCs or even prevent their formation from cellular origins.

Immune checkpoints are involved in a negative feedback mechanism that counteracts the activation signals in T cells to temper the immune response and maintain hemostasis in order to minimize tissue damage [164,165]. However, this mechanism is usually hijacked by tumor cells to escape immune surveillance, leading to tumor progression [166]. Immune checkpoint blockade (ICB) therapy is developed with the idea of harnessing the immune system to strengthen anti-tumor responses, which has achieved great success in the past decade [164,166]. In particular, immune checkpoint antagonists, such as monoclonal antibodies specific for PD-1, PD-L1, and CTLA-4, have revolutionized cancer therapy. Nevertheless, high levels of intratumoral heterogeneity and acquisition of an immunosuppressive TME (i.e., immune “cold” tumor) appear to be natural obstacles that have prevented ICB therapies from yielding satisfying outcomes in many human cancer types, including hormone-related cancers [167,168]. In breast cancer, triple-negative breast cancer (TNBC) is a subtype of breast cancer that often exhibits more lymphocyte infiltration in primary tumor sites than other subtypes. Due to their nature of generally higher immunogenicity, they are candidates for ICB-based immunotherapy, yet anti-PD-1/PD-L1 monotherapy only resulted in a mild response in TNBC patients [169,170]. In ovarian cancer, high-grade serous ovarian cancer (HGSOC) is the most common and lethal subtype. Due to their endogenous immunity at the molecular or T cell level, they can potentially be candidates for ICB-based immunotherapy as well, yet, to date, the outcome has fallen short of expectations [171]. Possibly due to the low mutational load and defects in T-cell-mediated anti-tumor immunity, prostate cancer is a type of cancer that is largely immune “cold” and responds to ICB-based immunotherapy poorly, with ICB clinical trials showing disappointing results for CRPC patients [172,173,174].

Expression analysis also showed that cancer stemness was strongly associated with cell-intrinsic suppression of endogenous retroviruses and type I IFN signaling and increased expression of multiple therapeutically accessible immunosuppressive pathways [161]. One strategy to improve responsiveness to ICB-based immunotherapy is to restore/enhance inflammation of the tumor (e.g., by activating IFN signaling and/or blocking immunosuppressive pathways). As discussed above, many TNBCs, HGSOCs, and prostate cancers may originate from their corresponding LPs (in the case of HGSOC, FT secretory stem/progenitor cells). TICs/CSCs of these cancer types may inherit the innate immune program (e.g., TLRs) from their cellular origins; if so, this may offer a unique opportunity to stimulate/enhance immune reactions in them (e.g., by activating their innate immune program using TLR agonists), making them more amenable to ICB-based immunotherapy (Figure 2). In support of this idea, in a recent study using a breast cancer metastasis model, *MMTV-PyMT*, it was shown that combined use of monophosphoryl lipid A (MPLA) and IFNγ reduced primary tumor growth and metastasis by activating a collaborative innate-adaptive immune response [175]. MPLA is a TLR4 agonist modified from lipid A, the biologically active part of Gram-negative bacterial LPS endotoxin; compared to LPS, MPLA exerts similar immunostimulatory activity but with reduced toxicity and has been approved by the FDA to use against cancer-causing HPV [77,176,177]. We showed previously that *MMTV-PyMT* mammary tumors may originate from mammary alveolar LPs [35], which express TLR pathway genes (e.g., *Tlr4*, *Cd14*, *Lbp*) [178]. This raises an intriguing possibility that the effectiveness of this approach was in part mediated via activation of TLR signaling by MPLA in *PyMT* TICs/CSCs, which may have inherited the TLR/innate immune program from LPs. The therapeutic potential of targeting TLR pathways in breast and prostate cancers was also discussed in other reviews [77,179]. Similar to our idea here (which focuses on targeting TLR^+^ TICs/CSCs), TLR agonists have also been viewed as either a possible therapeutic agent or as a vaccine adjuvant toward cancers [180]. Of note, in a transplantation-based ovarian cancer mouse model (i.e., ID8 model), TLR4 agonist LPS was tested to determine whether activation of TLR4 signaling could reshape the cancer immune signature [181]; even though this treatment did not result in survival benefit, it should be pointed out that the ID8 model originated from OSE cells (rather than FTE cells) [182], which do not express TLR pathway-related genes [75]. This study thus highlights the potential importance of choosing the right cancer for testing this strategy; that is, the strategy of boosting ICB-based immunotherapy via activation of TLR signaling might be most effective for cancer cells (or their TIC/CSC subset) equipped with the TLR/innate immune program. In addition, due to the dual roles of TLR pathways in cancer development [100], caution should be taken when using TLR agonists so that a window of opportunity is chosen to maximize the efficacy of immunotherapy and minimize any potential pro-tumor activities (e.g., chronic inflammation, inflammation-associated tissue repair).

## 8. Conclusions

In the last 10–15 years, studies in mouse models and human tumors have shown that progenitors for “secretory” cells (i.e., milk-producing alveolar cells, prostate luminal cells, FT secretory cells) are the preferred cellular origins of several common hormone-related cancers (i.e., breast, prostate, and ovarian cancers). A common feature of these LPs is that they all express genes related to the innate immune pathways (e.g., TLR pathways). Recent demonstration of the presence of microbes (e.g., bacteria) in their corresponding normal tissues and cancers suggests potential activation of innate immune responses in these LPs, which may contribute to initiation and progression of their corresponding cancer types. This can be achieved by chronic inflammation, LP-mediated epithelial tissue repair triggered by inflammation-related tissue damage, and crosstalk between evolving TICs/CSCS and immune cells (e.g., macrophages). Potential inheritance of the innate immune program by TICs/CSCs from their cellular origins may convey unique vulnerability to these TICs/CSCs, which may be harnessed for their elimination by enhancing immunotherapy. Although we focus on a discussion of hormone-related cancers in this review, we suspect that the same concept may also be applicable to other human epithelial cancer types that involve exposure to microbes (e.g., lung cancer, intestine/colon cancer).
